# Contradictions in Chilean public education policy: physical education and health case

**DOI:** 10.3389/fspor.2024.1410849

**Published:** 2024-07-12

**Authors:** Alixon David Reyes Rodríguez, Ezequiel Martínez Rojas

**Affiliations:** ^1^ Research Department, Universidad Adventista de Chile, Chillán, Chile; ^2^ Vice Rector’s Office for Research and Innovation, Universidad Arturo Prat, Iquique, Chile

**Keywords:** public policy, physical education, health, overweight, obesity

## Abstract

The purpose of this text is to point out the contradiction existing in Chilean educational public policy regarding the curricular reform that converted the subjects of Physical Education and Health, History, Arts, and Religion into optional subjects for the 3rd and 4th years of high school. As far as Physical Education is concerned, scientific evidence shows that such conversion to the status of optional subject goes against the policies of other executive ministries and against the possibilities of reversing the statistics that show Chile as one of the countries in America with the highest percentage of overweight and obese children and adolescents.

## 1 Introduction

In recent years, the paradigm of public policy as an integrated and multidimensional instance has been gaining strength (1), and it seems to be understood today that a public policy generated and attended to from the particularity and the disciplinary or technocratic solitude, whatever the field, is a thing of an increasingly distant past. Along with this, evidence-based public policy is also a current paradigm that occupies public attention, and points to the correspondence and coherence between political decisions, scientific evidence, contexts and national, regional and local realities. Although it is true that the latter paradigm has some disadvantages (2), “scientific evidence should be above any *a priori* consideration regarding of the goodness of a public management model” (3), in addition to the fact that it is necessary to understand that public policy is multifactorial, and that it is enriched by several approaches. approaches, trends, contexts, scenarios, regulatory frameworks, the telos of the policy itself, and without having to depend exclusively on scientific evidence, it does not seem a wise decision to dispense with it either (4, 5). If the question is asked about the number of studies that can be scientific evidence, and of what type, well, this could be answered by stating that the number of studies will be determined by the saturation points of the same evidence, and the typology, by its diversity and epistememethodological quality (5).

The object of this analysis reports an educational policy in which there is a curricular reform for secondary education notified in Agreement N° 057/2019 (6), and which implies that the subject of Physical Education and Health, together with those of History, Arts and Religion, became optional subjects from the third year of secondary education in Chile [See Table 1]. This decision has materialized important political and professional contradictions, that are not minor.

**Table 1:** General education curriculum, secondary education, Chile.

Common general education plan						
Subject		TP Hours per week		HC Hours per week		Artistic Hours per week
		With JEC	No JEC	With JEC	No JEC	With JEC
Language and literature		3 h	3 h	3 h	3 h	3 h
Mathematics		3 h	3 h	3 h	3 h	3 h
Civic education		2 h	2 h	2 h	2 h	2 h
Philosophy		2 h	2 h	2 h	2 h	2 h
English		2 h	2 h	2 h	2 h	2 h
Science for citizenship		2 h	2 h	2 h	2 h	2 h
Common elective general education plan^a^						
Religion	History, geography and social sciences	(2 h)^a^	(2 h)^a^	2 h	2 h	2 h
	Arts					
	Physical education					
Total		14 h	14 h	16 h	16 h	16 h
Plan diferenciado						
Technical-professional		22 h	22 h	–	–	–
Humanistic-scientific		–	–	18 h	18 h	–
Artistic		–	–	–	–	21 h
Hours of free disposal						
		6 h	2 h	8 h	2 h	5 h
Total		42 h	38 h	42 h	36 h	42 h

Source: Library of the National Congress of Chile.

TP, General Education Technical-Professional; HC, General Education Humanistic-Scientific; JEC, full day equivalent.

^a^ Common general elective training plan corresponding to the common general training plan.

## 2 Political implications

The decision of the Ministry of Education (MINEDUC) argues that the curriculum has not been weakened as long as the aforementioned subjects do not disappear, but rather, from the 3rd and 4th grades, they become optional among a wide range of other options, responding to students' interests and vocations (7). The measure was applied as of the 2020 school year, despite the fact that, in 2019, a request for the annulment of such measure was presented (8), and, despite the fact that bodies and institutions such as the College of Teachers of Chile, the National Academic Council of Physical Education (CANEF), the College of Physicians, the Chilean Olympic Committee, the Institute of Nutrition and Food Technology, the ELHOC Research Group “Epidemiology of Lifestyles in Chile”, in short, a good part of the academic and scientific community in Chile produced public documents arguing with scientific evidence and requesting a reversal of the measure (9–12).

According to the Ministry of Education (13), the measure places Chile within the framework of a worldwide trend, in which, according to the educational authority, young people in 3rd and 4th year of secondary education have greater possibilities of vocationally choosing their training in the last years of schooling. In addition to this, there is the known deficit (and the projection of such deficit) of Physical Education teachers in Chile, as well as in other areas (14–16), and that the institutional framework does not have close possibilities of reducing existing gaps in terms of facilities, equipment, material-teaching resources, the decrease of applicants in pedagogy careers (17), among other elements and reasons put forward by different sectors that support the ministerial measure.

The School of Physical Education of the Pontificia Universidad Católica de Valparaíso (18), in a public statement, said:

It is paradoxical and somewhat disappointing that this CNED agreement goes in the opposite direction to the presidential discourse of the current government, which has expressed its willingness to advance public policy, in order to contribute to the development of physical activity and sport in all social strata (sec. 1/1; p. 4).

Now, why is there a contradiction in Chilean public policy in this particular case? Well, the scientific evidence shows vehement data that question such a decision, specifically in relation to the conversion of the subject of Physical Education and Health as an optional from the 3rd and 4th year of secondary education in Chile. Evidence that, in addition, questions the inconsistent way in which public policy was generated without consultation, despite the fact that in Chile there are support instances such as the *Evidence-Informed Health Policies Unit* (19).

Although, on the one hand, progress was made with the implementation of policies such as the programs “Elige Vivir Sano”, “Elige Vivir sin Drogas”, “Crecer en Movimiento”, “Política Nacional de Actividad Física 2019–2025”, “Plan Nacional de Actividad Física Escolar”, on the other hand, making the subject optional for the courses indicated, generates a contradiction at the level of public policies, and it implies a significant setback.

Scientific evidence shows that, although it is true that the time dedicated to the school Physical Education class is not enough to cover the minimum physical activity recommendations of organizations such as the World Health Organization (20), without their help it will be impossible to reverse the statistics that indicate that Chile is one of the countries with the greatest problems of childhood obesity in America (21, 22). In addition, the benefits, not only of physical exercise in children's health, but also of its promotion from the Physical Education class for the adoption of healthy lifestyles, are already well documented (23–26). “The more hours of Physical Education per week, the higher the levels of physical activity” (p. 2) (27).

In 2018, Chile reported the penultimate place among 49 countries in terms of physical activity in children and young people (21). It is known that 3 out of ten children under 5 years of age perform at least 3 h of physical activity per day in Chile, and 2 out of ten adolescents register 1 h per day (28). Between 12.8% and 33% of Chilean students participate 3 or more times per week in Physical Education and Health class, while 1 in 4 students present positive reinforcement (29). This latest study reveals that 80% of school-aged children are physically inactive, and as children grow older they perform less physical activity (30), having that, in 2016, the prevalence of physical inactivity in children between 11 and 17 years old was in the order of 84.2% in Chile, and in girls, 91.2%.

The *Childhood Obesity Radiography* (31) shows that obesity grew in Chile by 66.3% between 2005 and 2018 in children under six years of age; between 1997 and 2018, overweight and obesity in children in 1st grade increased by 50.9%; while in 1st grade children it increased by 46.4% between 2009 and 2018. In Chile: “In the case of adolescents, the prevalence of obesity will increase from 13.5% to 19.8% between 2016 and 2030” (32). In addition, 58.3% of school children in Chile present excess malnutrition (overweight + total obesity) (33), “with Chile being one of the four countries with the highest prevalence of childhood overweight and obesity in the American continent” (34). Chile was, along with Qatar, the country with the highest combined prevalence of thinness and obesity in school-aged children and adolescents in 2022 (22).

Students in 3rd and 4th grade have the highest prevalence of physical inactivity in Chile, exceeding 80% in both men and women (22, 34). By 2020, 38% of children in Chile had a high body mass index (BMI), and it is estimated that by 2025, that percentage will increase to 49% (35).

Studies reinforce the stated need for the increase of weekly hours for the compulsory Physical Education subject (36–38). An example of this can be found in countries such as Venezuela, where the subject now occupies three weekly class sessions with six mandatory hours in total, which is added to the elective training areas of Physical Activity, and Leisure and Recreation, which can add up to two hours per week each and can be chosen simultaneously, which would even allow increasing the hours to 10 h per week available for the promotion of physical exercise and adoption of healthy lifestyles (39, 40). In the case of Mexico, the policy of quality Physical Education aims at increasing weekly hours of Physical Education (41). In countries such as Colombia, Chile, Spain, Portugal, Dominican Republic, the need to increase weekly hours for school Physical Education is reinforced (37), reinforcing the guidelines of the World Health Organization (42).

Questions arise about what has been reported, is: how is it that the MINEDUC decided to make the subject of Physical Education and Health optional for the last two years of Chilean secondary education, when the evidence shows data that place Chile as one of the countries in the Americas with the highest rate of obesity and overweight in the child and adolescent population, and with one of the most worrisome projections in the region?, how is it possible to ignore the exercise of public policy that contradicts the efforts of other ministries of the national executive?

Reversing such a measure is sensible. There's still time. Given that public policy requires scientific evidence, among other indicative sources of information, being coherent in public management is an urgency in any country (5). Hence, it is considered that the public policy considered has not been coherent, and contradicts the data presented by the scientific evidence, even though Chile uses the paradigm of evidence-based public policy to guide decision-making.

An evidence-based educational policy must be based on rigorous research that allows to sustain educational practices aimed at achieving reliable results, in such a way that decisions should not be based on the ideology of the government of the day, but on effectiveness in solving problems, thus seeking that society demands the continuity of those successful programs. Even if the government is changed (4).

## 3 Practical recommendations

Among the recommendations that can be considered are: (a). creation of a National Advisory Council on Physical Education, and its integration in working sessions with the Ministry of Education; (b). national consultation with teachers and specialists in the field; (c). review of scientific evidence and constitution of a body of considerations pointing out the incongruence of Agreement No. 057/2019; (d). work and analysis meetings with the Curriculum and Evaluation Unit, the General Education Division and the Center for Improvement, Experimentation and Pedagogical Research of the Ministry of Education, in order to generate the in-depth review of the educational public policy that defines the electivity of the subject of Physical Education for 3rd and 4th year of high school; (e). reversal of this policy by making Physical Education compulsory again; (f). increase of hours per week for Physical Education in the Curriculum; (g). articulated work between the Ministry of Education, the Ministry of Sports and the Ministry of Health in the definition of policies public associated with physical exercise, sports and recreation as public health issues; (h). building the national network of schools promoting healthy lifestyles.

In relation to potential challenges that could affect the implementation of the recommendations provided, the following could be considered: financial costs that would involve reversing Agreement No. 057/2019 insofar as it implies that Physical Education teachers should cover the subject on a mandatory basis raising the annual budget of MINEDUC, and to this is added the necessary investment in terms of infrastructure and equipment; curricular reformulation that would involve slowing down administrative processes at the national level, and that would imply the adhesion of other areas that, like Physical Education, were also affected by the measure (e.g.,.: History and Geography, Arts); different agendas of the actors that could be participating in ministerial decision-making; political cost of a possible reversal of Agreement No. 057/2019, inasmuch as it is understood that a measure of such magnitude implies political agreements between different sectors of national life.

## 4 Conclusions

There are several elements that appear to be key to the public policy framework. As has already been pointed out, articulation in the generation, development, management, execution and evaluation of public policy is, as of today, a sine qua non condition for governance. Disciplinary, technical and political solitude must have counterproductive effects which, precisely because they are public policies, have an effect on the lives of thousands and millions of people who are affected by the implementation of programs and policies that have not been sufficiently dialogued, articulated and brought together in a multiplicity of responsible authors. Secondly, it is necessary to understand that, although it is true that scientific evidence cannot constitute the first and only element for decision making, it does not seem sensible, either, to ignore what it has to dictate. And, in Chile, both things have happened in the context of the approval of Agreement No. 057/2019, that is: (1). it ignores what scientific evidence dictates by erecting a public policy that condemns just the population with greater vulnerability in terms of physical inactivity, overweight and obesity; and, (2). the decision is made by a public policy, ignoring the articulation with other instances that go in opposite ways to the decision taken by the ministry responsible for education in the country. In Chile there are interlocutors who, despite the time, are able and willing to dialogue and collectively build new spaces, scenarios and regulatory provisions to correct the mistake.

International experience shows that the trend in Latin America is to recognize the need to increase the number of hours per week for physical education in schools. Mexico, Colombia and Ecuador have already incorporated statements in this regard in their curricular guidelines. And, moreover, given that the argument given by MINEDUC in Chile focuses on the vocational experience of young people, there are examples such as the Venezuelan case in which the vocational experience has been focused as a priority in secondary education, but not at the cost of Physical Education, but, on the contrary, there is evidence of an increase in the sessions and weekly hours of compulsory Physical Education, even creating vocational training areas for physical activity, sport and recreation.

The school should be a space that promotes healthy lifestyles, the adoption of healthy habits that allow the adherence to physical exercise, not as a hedonistic pattern, but as a public health issue. And public policy should accompany such efforts, from the curriculum and from other extracurricular spheres.

## Data Availability

The original contributions presented in the study are included in the article/Supplementary Material, further inquiries can be directed to the corresponding author.
